# Effects of Nonpharmaceutical COVID-19 Interventions on Pediatric Hospitalizations for Other Respiratory Virus Infections, Hong Kong

**DOI:** 10.3201/eid2801.211099

**Published:** 2022-01

**Authors:** Susan S. Chiu, Benjamin J. Cowling, J.S. Malik Peiris, Eunice L.Y. Chan, Wilfred H.S. Wong, Kwok Piu Lee

**Affiliations:** The University of Hong Kong, Hong Kong, China (S.S. Chiu, B.J. Cowling, J.S. Malik Peiris, E.L.Y. Chan, W.H.S. Wong);; Pamela Youde Nethersole Eastern Hospital, Hong Kong (K. Piu Lee)

**Keywords:** COVID-19, 2019 novel coronavirus disease, coronavirus disease, severe acute respiratory syndrome coronavirus 2, SARS-CoV-2, viruses, respiratory infections, zoonoses, nonpharmaceutical interventions, pandemic, respiratory virus hospitalization, pediatrics, Hong Kong

## Abstract

To determine the effects of nonpharmaceutical interventions (NPIs) for coronavirus disease on pediatric hospitalizations for infection with respiratory viruses other than severe acute respiratory syndrome coronavirus 2, we analyzed hospital data for 2017–2021. Compared with 2017–2019, age-specific hospitalization rates associated with respiratory viruses greatly decreased in 2020, when NPIs were in place. Also when NPIs were in place, rates of hospitalization decreased among children of all ages for infection with influenza A and B viruses, respiratory syncytial virus, adenovirus, parainfluenza viruses, human metapneumovirus, and rhinovirus/enterovirus. Regression models adjusted for age and seasonality indicated that hospitalization rates for acute febrile illness/respiratory symptoms of any cause were reduced by 76% and by 85%–99% for hospitalization for infection with these viruses. NPIs in Hong Kong were clearly associated with reduced pediatric hospitalizations for respiratory viruses; implementing NPIs and reopening schools were associated with only a small increase in hospitalizations for rhinovirus/enterovirus infections.

Coronavirus disease (COVID-19), caused by severe acute respiratory syndrome coronavirus 2 (SARS-CoV-2), was first detected in Wuhan, China, in late 2019. The World Health Organization (WHO) declared the outbreak to be a pandemic on March 12, 2020. In Hong Kong, where residents share the collective trauma of the severe acute respiratory syndrome outbreaks in 2003, masking was spontaneously and voluntarily adopted by residents in late January 2020 when news of an outbreak of pneumonia with high illness and death rates in mainland China reached Hong Kong. Primary and secondary school class suspension immediately followed. Other measures of social distancing were also put in place. Such nonpharmaceutical interventions (NPIs) were associated with reduced transmission of SARS-CoV-2 in Hong Kong (*1*).

The Hong Kong Special Administrative Region is made up of Hong Kong Island, Kowloon Peninsula, the New Territories, and some sparsely populated outlying islands. The only 2 public hospitals under the Hospital Authority on Hong Kong Island are Pamela Youde Nethersole Eastern Hospital (PYNEH) and Queen Mary Hospital (QMH), which manage 71.1% of all pediatric admissions on the island (*2*). Since 2003, we have captured hospitalizations of children with confirmed virus infections in these 2 hospitals and documented population-based hospitalizations resulting from infections with respiratory pathogens in Hong Kong (*3*–*7*). Our objectives with this study were to document the effects of NPIs on pediatric hospitalizations resulting from respiratory virus infections in Hong Kong in the first 12 months of the COVID-19 pandemic and the additional effects of school closure when other NPIs were implemented.

The study protocol was approved by the joint Institutional Review Board of the University of Hong Kong and the Hospital Authority Hong Kong West Cluster, as well as the Hospital Authority Hong Kong East Cluster Research Ethics Committee. The need for written consent was waived because testing for respiratory pathogens is standard and routine for all children admitted for an acute respiratory infection in the 2 study hospitals.

## Methods

### Study Design

All children admitted to PYNEH and QMH with an acute febrile illness or respiratory signs/symptoms are tested by multiplex PCR for influenza A virus, influenza B virus, respiratory syncytial virus (RSV), adenovirus, parainfluenza virus types 1–4, human metapneumovirus (HMPV), and rhinovirus/enterovirus. Because viral respiratory infections account for >70% of acute febrile illnesses in children and because very young children with influenza virus infections may exhibit fever only, for the past 20 years these 2 hospitals have routinely tested children with an acute febrile illness for respiratory viruses at the time of admission. For the purpose of infection control, children with acute respiratory symptoms without fever (e.g., asthma exacerbation) were also routinely tested at admission. We captured the testing results of all such patients from a computerized medical system of the Hospital Authority, along with individual patient data on age, sex, and date of admission. For comparison, we also captured information about admissions for urinary tract infection (UTI).

### Statistical Analyses

We estimated hospitalization rates overall and stratified them by virus and patient age group. We also estimated the proportion of children for whom a respiratory virus was detected by multiplex assay each year. Estimated hospitalization rates and 95% CIs for each age group were based on the Poisson distribution. The numerator in each incidence rate was the number of laboratory-confirmed admissions in that age group, and the denominator was the estimated person-years at risk each year (estimated from the population of Hong Kong Island in that age group, according to the latest census of 2016) (*2*). Census data for infants <6 months of age were lacking, and we used as the denominator half of the population <1 year of age, assuming a constant birth rate over the year. We calculated age-specific population-based hospitalization rates for each virus by using the reciprocal of the proportion of children served by the 2 hospitals (71.1% in 2019). To adjust for a median incubation period of these respiratory viruses, we used a lag time of 4 days between school holidays/closures or reopening and hospitalization. We excluded from analysis children admitted in 2020 and 2021 for COVID-19 because a substantial proportion of them had imported cases, most of them identified during contact tracing, and the patients were admitted for isolation purposes, which was not the objective of our study.

We compared age-specific hospitalization rates for respiratory viruses for the 12 months after implementation of NPIs at the end of January 2020 with rates from the previous 3 years by using a Poisson regression model and adjusting for age and seasonality. To explore the contribution of school closures to reducing spread of respiratory viruses, we compared rates of hospitalization for acute febrile illness/respiratory symptoms and the proportion of viral infections detected during periods of school closure in 2020 and 2021 with that during school reopening under additional school-based infection control measures, accounting for the overall effect of NPIs during 2020–2021 and adjusting for age and seasonality. In the analysis for the additional effects of school reopening, we excluded viruses for which <10 hospitalizations were recorded in the NPI period February 2020–January 2021.

## Results

**Figure 1:**
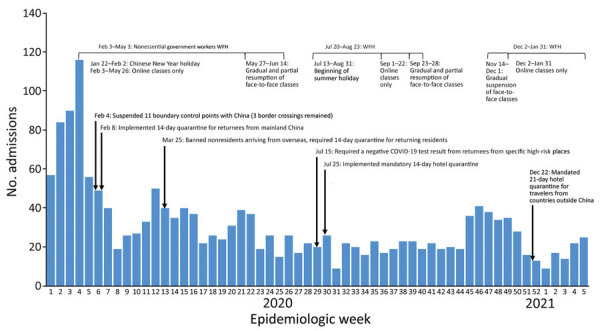
Acute hospitalization pediatric admissions for fever/respiratory symptoms at Pamela Youde Nethersole Eastern Hospital and Queen Mary Hospital, Hong Kong Island, China, and timeline of major interventions implemented by the government in response to COVID-19 in Hong Kong. COVID-19, coronavirus disease; WFH, work from home.

In Hong Kong, local schools close for the Chinese New Year, and in 2020, the holiday started on January 22. After the first imported case from China was detected on January 21, 2020, and with an increasing number of imported cases, on January 26, the Hong Kong government raised the Preparedness and Response Plan to emergency level and extended the Chinese New Year holiday of secondary schools, primary schools, kindergartens, childcare centers, and special schools; face-to-face instruction was not resumed until the end of May (https://www.info.gov.hk/gia/general/202001/26/P2020012600087p.htm) (Figure 1). Almost immediately after the government announcement, the Hong Kong population nearly universally and voluntarily wore surgical masks when outside of their homes (*1*), although the government initially advised against this and only later advocated mask use for persons with respiratory signs/symptoms according to recommendations of the World Health Organization and the US Centers for Disease Control and Prevention. The public also increased the practice of hand hygiene, and shops and restaurants required temperature checks before entry. Interventions implemented by the government enforced at various times and to different extents during the year included bans on public indoor or outdoor gatherings of >4 persons, travel restrictions limiting entry to Hong Kong residents only, mandatory quarantine for returnees, flexible work arrangements, and banning of in-restaurant dining to different extents. Other measures included cancelling large-scale events and closing theme parks, leisure, and cultural facilities (e.g., museums, sports centers, libraries). Religious services, concerts, conferences, and local mass gatherings were also cancelled.

**Figure 2:**
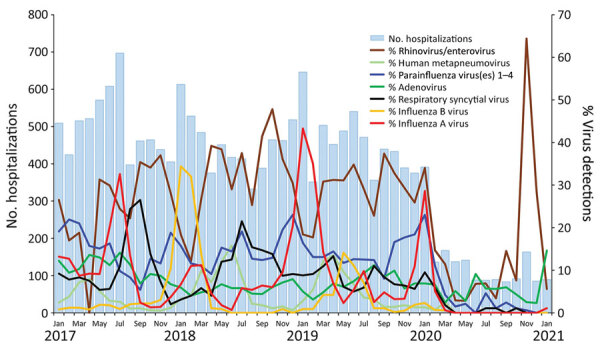
Pediatric hospitalizations for acute fever/respiratory symptoms and detection rates for respiratory viruses at Pamela Youde Nethersole Eastern Hospital and Queen Mary Hospital, Hong Kong Island, China, 2017–2021.

Annual hospitalizations of children for acute febrile illness/respiratory symptoms at PYNEH and QMH were 5,400–6,000 during 2017–2019 but were greatly reduced to 1,525 in 2020 (including 391 admissions in January before NPIs were implemented). Adjusting for age and seasonality, we estimated that rates of hospitalization for acute febrile illness/respiratory symptoms declined by 75% overall (95% CI 74%–77%). In addition, such admissions in 2020 were less likely to be associated with a respiratory virus (Table 1). For comparison, admissions for UTI were 146 in 2017, 162 in 2018, 133 in 2019, and 120 in 2020. Until January 2020, before implementation of NPIs, 74.4% of hospitalizations for acute fever/respiratory symptoms were associated with a virus, but a virus was detected in only 8.8% of similar patients hospitalized in April and 7.1% in May of that year. Almost no children were hospitalized for influenza A or B virus infection after NPIs were put in place (Table 1; Figure 2). Rates of influenza A pediatric hospitalizations had increased in February and March every preceding year, but in February and March 2020, pediatric hospitalizations for influenza A infection almost disappeared. In January 2021, only 19 children were admitted for acute febrile illness/respiratory symptoms, and influenza A was detected in 1, rhinovirus/enterovirus in 5, and adenovirus in 13. In Hong Kong, RSV infections usually led to hospitalization almost year-round, but in 2020, hospitalizations for RSV nearly disappeared. The rate of virus detection was highest in November, when schools fully reopened; virus detection was mainly rhinovirus/enterovirus.

**Table 1:** Pediatric hospitalizations for acute febrile illness or respiratory symptoms caused by viruses other than severe acute respiratory syndrome coronavirus 2 and numbers of infections detected, Pamela Youde Nethersole Eastern Hospital and Queen Mary Hospital, Hong Kong Island, China*

Month	No. laboratory-confirmed virus infections/no. hospitalizations for acute febrile illness or respiratory symptoms (%)				
	2017, n = 6,010	2018, n = 5,445	2019, n = 5,44	2020, n = 1,525	2021, n = 89
Jan	349/509 (68.6)	457/613 (74.6)	514/646 (79.6)	291/391 (74.4)	19/89 (21.3)
Feb	269/424 (63.4)	377/528 (71.4)	259/351 (73.8)	46/136 (33.8)	NA
Mar	332/515 (64.5)	346/484 (71.5)	389/503 (77.3)	31/167 (18.6)	NA
Apr	351/521 (67.4)	229/375 (61.1)	327/452 (72.3)	12/137 (8.8)	NA
May	394/571 (69.0)	313/451 (69.4)	339/488 (69.5)	10/141 (7.1)	NA
Jun	456/608 (75.0)	271/417 (65.0)	378/540 (70.0)	13/88 (14.8)	NA
Jul	557/697 (79.9)	308/413 (74.6)	308/471 (65.4)	14/87 (16.1)	NA
Aug	280/397 (70.5)	190/332 (57.2)	195/356 (54.8)	10/89 (11.2)	NA
Sep	316/461 (68.5)	259/388 (66.8)	270/439 (61.5)	18/83 (21.7)	NA
Oct	293/464 (63.1)	330/464 (71.1)	267/433 (61.7)	13/91 (14.3)	NA
Nov	265/438 (60.5)	333/462 (72.1)	219/389 (56.3)	110/163 (67.5)	NA
Dec	264/405 (65.2)	396/518 (76.4)	227/375 (60.5)	26/85 (30.6)	NA
Median (range), %†	67.9 (60.1–79.9)	71.2 (57.2–76.4)	67.4 (54.8–79.6)	17.3 (7.1–74.4)	NA

*Respiratory viruses were influenza A virus, influenza B virus, respiratory syncytial Virus, adenovirus, parainfluenza virus types 1–4, human metapneumovirus, enterovirus/rhinovirus. NA, not applicable. †Kruskal-Wallis test demonstrated significant difference among different years (p<0.001), and Dunn post hoc test indicated a significant difference for only 2020 compared with previous years (p<0.05).

Comparison of age-specific hospitalization rates associated with each respiratory virus in the 4 years studied showed that rates were greatly reduced in 2020 (Appendix). In general, among children with infection with any virus, hospitalization rates were higher among children <5 years of age than among older children; however, hospitalization rates were also reduced in 2020. Specifically, among children <1 year of age, rates of reduction were 82% for influenza virus A, 100% for influenza virus B, 93% for RSV, 79% for adenovirus, 85% for parainfluenza viruses, 86% for HMPV, and 84% for rhinovirus/enterovirus. A regression model adjusted for age and seasonality showed that hospitalization rates for respiratory viruses declined by 85%–99% (Table 2). When we further analyzed the difference in hospitalization rates during the period when schools were reopened in 2020–2021 under school-based infection control measures compared with the periods in 2020–2021 when they were closed, we found an increased rate of pediatric hospitalizations for rhinovirus/enterovirus only, not for other viruses.

**Table 2:** Relative reductions in incidence rates of pediatric hospitalizations during period of NPI and incidence rate ratio during school reopenings compared with school closure periods, Hong Kong Island, January 2017–January 2021*

Virus	Relative reduction, % (95% CI)†	Incidence rate ratio (95% CI)‡
Influenza A	99 (98–100)	NE
Influenza B	99 (97–100)	NE
Respiratory syncytial virus	98 (97–99)	NE
Adenovirus	85 (80–88)	1.27 (0.85–1.89)
Parainfluenza types 1–4	96 (95–97)	1.08 (0.85–1.69)
Human metapneumovirus	98 (95–99)	NE
Rhinovirus/enterovirus	87 (85–89)	1.72 (1.37–2.17)

*NE, not estimated because <10 hospitalizations with the given virus were recorded during the NPI period; NPI, nonpharmaceutical intervention. †Estimated as 1 minus the incidence rate ratio in a Poisson regression model for virus-specific hospitalization rates, adjusted for age and calendar time, comparing the period February 2020–January 2021 (NPI period) with January 2016–2020. ‡Estimated in a Poisson regression model for virus-specific hospitalization rates, comparing the period when schools were reopened with infection control measures versus when they were closed during 2020–21, adjusted for the overall risk reduction during the NPI period as well as for age and calendar time. An incidence rate ratio >1 indicates an increased rate during the school resumption period and vice versa.

## Discussion

Our study documents a drastic reduction in hospitalizations for acute respiratory virus disease in children and adolescents during a 12-month period when NPIs were in place in Hong Kong at the beginning of the COVID-19 pandemic. Using UTI hospitalizations as a control, for which hospitalizations in 2020 were about the same as in previous years, we cannot conclude that the reduced hospitalizations for acute febrile illness/respiratory symptoms and respiratory viruses in 2020 resulted from avoidance of public hospitals during the COVID-19 pandemic. Infants <1 year of age should be the least affected directly by school closure and social distancing measures; however, hospitalization for respiratory viruses decreased by 79%–100% for this age group, illustrating the role of household transmission of these respiratory viruses from older siblings and adults.

Reduced infections during the COVID-19 pandemic have been reported around the world (*8*–*13*). In France, where public health interventions for the pandemic included school closures and national lockdown, a >70% decrease was found for pediatric emergency department visits and admissions for acute gastroenteritis, common colds, bronchiolitis, and acute otitis media (*8*). Researchers reported a >99% reduction of RSV cases detected at sentinel laboratories in Belgium during the usual RSV season of 2020 (*9*). In winter 2020 in Western Australia, detection of RSV infection in children decreased by 98% and influenza by 99.4%, despite reopening of schools (*10*). The authors thought that international and even national border restrictions might have played a role by preventing external introductions of these viruses; however, that analysis did not include hospital admission data and the report did not mention the extent of mask use in the society and in schools. In Australia, New Zealand, the United States, Chile, and South Africa, which reported reduced detection of influenza and other respiratory virus, NPI measures had included stay-at-home orders or lockdowns (*11*–*13*). Hong Kong never implemented a full lockdown or stay-at-home order, although avoiding crowding was recommended to the public. A district where residents had to undergo mandatory testing implemented a mandatory 48-hour lockdown involving several blocks, but that was on January 23, 2021, and had a negligible effect on our findings. When the most stringent measures were implemented, in addition to school closures, the Hong Kong government closed its offices for nonemergency services and encouraged other public institutions and the private sector to adopt working from home, which was followed to different extents. Shops and restaurants remained open, although in-restaurant dining after 6:00 pm was banned in July and December 2020.

According to a study of the first 3 months of 2020 in Hong Kong, influenza transmission declined substantially after NPIs were instituted in late January, from an estimated effective reproduction number of 1.28 (95% CI 1.26–1.30) before the start of school closures to 0.72 (95% CI 0.70–0.74) during the closure weeks (*1*). In our study, we found that as NPIs were continued with varying stringency at different times according to the number of local and imported cases of SARS-CoV-2 infections, reduced pediatric hospitalizations for influenza persisted throughout the rest of the year and into the first month of January 2021, and thus, there was no influenza season during the winter of 2020–21. However, the drastic reduction in pediatric hospitalizations for acute viral respiratory infections was probably not attributed mostly to school closure when other NPIs, including international travel restrictions, were in place. 

Before 2020, hospitalizations and virus detection were lower during summer holidays compared with other times of the school year, but they were still significantly higher than they were in 2020 with NPIs in place, regardless of school closure. When other NPIs were being used, mean rates of hospitalization for most respiratory viruses were not further reduced by school closure (Table 2). Hospitalization rates for rhinovirus/enterovirus did, however, increase substantially, which could be associated with a loss of population immunity during the long school closure period (*14*). Resumption of school in 2020–21 was not a complete return to prepandemic conditions because students were required to wear face masks; schools typically only resumed for half days; and temperature checks, enhanced hand hygiene, and social distancing measures were used in the classrooms (*15*). Rhinovirus can spread by fomites, and evidence indicates that surgical masks may not be very effective for blocking rhinovirus transmission. Leung et al. recently showed that surgical face masks effectively reduced detection of influenza virus and coronaviruses, but not rhinovirus, in exhaled air (*16*). Nevertheless, the school-based infection control measures in the context of other NPIs in the community appeared sufficient to limit transmission of other respiratory viruses.

Our observation that school resumption did not affect rates of hospitalization for infection with other respiratory viruses, except rhinovirus, is consistent with modeling studies of SARS-CoV-2 that predicted that school closures alone would prevent only 2%–4% of deaths, much less than other social distancing interventions (*17*). Fong et al. also demonstrated absence of SARS-CoV-2 transmission in the school setting from the 20 students, 5–17 years of age, who had acquired SARS-CoV-2 infection from household contacts or from unknown sources (*15*). On March 18, 2020, the UN Educational, Scientific and Cultural Organization estimated that 107 countries had implemented national school closures in an effort to combat COVID-19, affecting half the global student population (*18*). By mid-April 2020, a total of 192 countries adopted national school closure policies, affecting ≈1.6 billion (>90%) of the world’s student population (*19*). School closure during the COVID-19 pandemic carries high social and economic costs across communities and has the most severe effect on the most vulnerable and disadvantaged populations (*19*,*20*). The harms associated with school closure are profound and far-reaching. Delineating the effect of school closure on the mitigation of COVID-19 among other concurrent NPIs has been attempted but is nearly impossible, and without such measures, no comparison with baseline was possible. This study of hospitalization for other respiratory viruses with modes of transmission presumably similar to those of SARS-CoV-2 provides insight into the role of school closure in the context of other NPIs and lends support for keeping schools open with additional school-based infection control measures in place during a pandemic when other NPIs can be implemented in the community.

The wearing of face masks in Hong Kong was extraordinary. First, rapid and nearly universal masking was initiated by the public ahead of government recommendations (*1*). Second, persons in Hong Kong wore surgical masks outside the home regardless of respiratory symptoms, which was not the initial government recommendation. The rate of mask use was remarkably high in the population, including among young children. A survey found that during the first 100 days of the COVID-19 outbreak, face mask use was >95%–97% in all 18 Hong Kong administrative districts (*21*). That study compared the incidence of COVID-19 in Hong Kong with that in selected countries with a well-established healthcare system (e.g., Asia, Europe, and North America), where face mask use was not universally adopted in the community, and concluded that communitywide mask wearing may contribute to the control of COVID-19. In our previous study that showed substantial influenza virus transmission in the first 3 months of the COVID-19 pandemic in Hong Kong, 98.2% of respondents surveyed during mid-February and mid-March reported wearing masks when going out (*1*).

Our first study limitation is that it was an observational study and that different NPIs overlapped and changed repeatedly throughout the year in Hong Kong, making it difficult to precisely attribute the exact effect of each intervention. In our discussion of the effects of school closure and school resumption, we had data only on pediatric hospitalizations and did not study the effects of school closures or resumptions on infections and hospitalizations of adults. We also did not have data on children with respiratory viral infections who did not need hospitalization.

One strength of our study is that data were drawn from a well-established system that captures hospitalizations for acute respiratory infection for which all patients have been routinely tested by PCR since 2003, so there was no selection bias for testing. There was also no data capture bias because data for all children hospitalized with an acute respiratory illness were captured throughout the years. Because these 2 hospitals provide hospitalization for 71% of the population on Hong Kong Island, we were able to provide age-specific population-based estimates of hospitalizations. In addition, Hong Kong usually has prolonged RSV infection and influenza seasons, and this 12-month study enabled us to document the lack of viral respiratory diseases in 2020.

In conclusion, our study showed that measures implemented at government and individual levels to control COVID-19 in Hong Kong substantially reduced pediatric hospitalizations for other respiratory viruses and that school closures were associated only with further reduction of hospitalizations (except for rhinovirus/enterovirus) when other NPIs were implemented. The NPIs used in Hong Kong in 2020 would probably effectively control future pandemics caused by respiratory viruses (e.g., influenza or coronaviruses) while vaccines were being developed, and schools should be considered an essential service that could be kept open with appropriate infection control measures in place.

Appendix Supplemental results for study of effects of nonpharmaceutical COVID-19 interventions on pediatric hospitalizations for other respiratory virus infections, Hong Kong.
