# Supplementary material for: Effects of Nonpharmaceutical COVID-19 Interventions on Pediatric Hospitalizations for Other Respiratory Virus Infections, Hong Kong
Source: Emerg Infect Dis. 2022 Jan;28(1):62–8. doi: 10.3201/eid2801.211099 (PMC8714236; doi:10.3201/eid2801.211099)
Supplement: Appendix — Supplemental results for study of effects of nonpharmaceutical COVID-19 interventions on pediatric hospitalizations for other respiratory virus infections, Hong Kong. [file 21-1099-Techapp-s1.pdf]

Article DOI: https://doi.org/10.3201/eid2801.211099

Effects of Nonpharmaceutical COVID-19 Interventions on Pediatric Hospitalizations for Other Respiratory Virus Infections, Hong Kong Island

Appendix

Appendix Table. Annual rate of pediatric hospitalizations for viral respiratory infection/10,000 residents, Hong Kong Island, China, 2017–2020*

	Hospitalizations (95% CI)							
Virus, year	<6 mo	6 mo –<12 mo	<1 y	1–<2 y	2–<5 y	5–<10 y	10–<15 y	15–<18 y
HK Island Population	4,180	4,180	8,360	9,136	27,102	46,470	40,137	27,930
Influenza A								
2017	117.8 (82.0–163.8)	191.8 (145.3, 248.5)	154.8 (124.8–189.8)	141.6 (114.2–173.7)	133.4 (117.6–150.7)	58.4 (50.5–67.3)	19.6 (14.8–25.5)	8.6 (5.0–13.7)
2018	53.8 (30.8–87.4)	74.0 (46.4–112.1)	63.9 (45.2–87.8)	84.7 (63.8–110.2)	102.8 (88.9–118.1)	31.2 (25.4–37.8)	7.7 (4.8–11.7)	4.5 (2.1–8.6)
2019	117.8 (82.0–163.8)	134.6 (96.2–183.3)	126.2 (99.2–158.2)	144.7 (116.9–177.1)	145.3 (128.8–163.4)	51.5 (44.0–59.8)	18.6 (13.9–24.3)	9.1 (5.4–14.3)
2020	16.8 (5.5–39.3)	40.4 (20.9–70.5)	28.6 (16.7–45.8)	29.3 (17.6–45.7)	23.4 (17.0–31.2)	7.6 (4.9–11.2)	3.9 (1.9–6.9)	2.0 (0.5–5.2)
Influenza B								
2017	6.7 (0.8–24.3)	26.9 (11.6–53.0)	16.8 (8.1–30.9)	16.9 (8.5–30.3)	19.7 (14.0–27.1)	16.0 (12.0–21.0)	4.6 (2.4–7.8)	2.5 (0.8–5.9)
2018	30.3 (13.9–57.5)	67.3 (41.1–103.9)	48.8 (32.7–70.1)	77.0 (57.1–101.5)	78.4 (66.4–91.9)	52.7 (45.1–61.1)	15.8 (11.5–21.1)	5.0 (2.4–9.3)
2019	13.5	26.9	20.2	21.6	29.1	30.6	12.6	5.5

	Hospitalizations (95% CI)							
Virus, year	<6 mo	6 mo –<12 mo	<1 y	1–<2 y	2–<5 y	5–<10 y	10–<15 y	15–<18 y
	(3.7–34.5)	(11.6–53.0)	(10.4–35.3)	(11.8–36.2)	(22.0–37.7)	(24.9–37.1)	(8.8–17.5)	(2.8–9.9)
2020	0 (0–12.4)	0 (0–12.4)	0 (0–6.2)	1.5 (0–8.6)	1.6 (0.3–4.6)	0.6 (0.1–2.2)	1.4 (0.4–3.6)	0.5 (0–2.8)
RSV								
2017	383.6 (316.4–460.8)	333.1 (270.7–405.6)	358.3 (311.8–409.8)	224.8 (189.8–264.3)	120.4 (105.4–136.9)	7.3 (4.7–10.8)	2.5 (1.0–5.1)	2.0 (0.5–5.2)
2018	353.3 (289.0–427.7)	249.0 (195.5–312.6)	301.1 (258.6–348.6)	161.6 (132.2–195.7)	113.7 (99.1–129.7)	8.8 (5.9–12.6)	1.4 (0.4–3.6)	0.5 (0–2.8)
2019	275.9 (219.4–342.5)	201.9 (154.1–259.9)	238.9 (201.2–281.6)	147.8 (119.7–180.5)	88.2 (75.5–102.5)	7.0 (4.4–10.4)	2.5 (1.0–5.1)	1.0 (0.1–3.6)
2020	33.6 (16.2–61.9)	13.5 (3.7–34.5)	23.6 (12.9–39.5)	18.5 (9.5–32.3)	10.4 (6.3–16.0)	1.5 (0.5–3.5)	0 (0–1.3)	0 (0–1.9)
Adeno								
2017	60.6 (35.9–95.7)	175.0 (130.7–229.4)	117.8 (91.8–148.8)	160.1 (130.8–194.0)	138.6 (122.4–156.2)	51.5 (44.0–59.8)	11.9 (8.3–16.6)	2.5 (0.8–5.9)
2018	20.2 (7.4–43.9)	90.8 (59.9–132.2)	55.5 (38.2–78.0)	115.5 (90.8–144.7)	75.8 (64.0–89.1)	15.4 (11.5–20.3)	2.1 (0.8–4.6)	0.5 (0–2.8)
2019	37.0 (18.5–66.2)	77.4 (49.1–116.1)	57.2 (39.6–79.9)	115.5 (90.8–144.7)	94.4 (81.2–109.2)	21.5 (16.8–27.1)	4.6 (2.4–7.8)	2.5 (0.8–5.9)
2020	6.7 (0.8–24.3)	43.7 (23.3–74.8)	25.2 (14.1–41.6)	36.9 (23.7–55.0)	14.5 (9.7–21.0)	3.6 (1.9–6.3)	1.4 (0.4–3.6)	0.5 (0–2.8)
Para 1–4								
2017	191.8 (145.3–248.5)	329.7 (267.7–401.9)	260.8 (221.3–305.2)	275.6 (236.7–319.0)	199.3 (179.9–220.2)	39.0 (32.6–46.4)	8.8 (5.7–12.9)	2.5 (0.8–5.9)
2018	171.6 (127.8–225.6)	265.8 (210.4–331.3)	218.7 (182.7–259.7)	250.9 (213.9–292.6)	193.1 (173.9–213.7)	38.1 (31.8–45.4)	5.6 (3.2–9.1)	2.0 (0.5–5.2)
2019	148.0	282.6	215.3	214.0	178.5	38.1	5.3	2.0

	Hospitalizations (95% CI)							
Virus, year	<6 mo	6 mo –<12 mo	<1 y	1–<2 y	2–<5 y	5–<10 y	10–<15 y	15–<18 y
	(107.6–198.8)	(225.4–349.9)	(179.6–256.0)	(179.9–252.7)	(160.2–198.4)	(31.8–45.4)	(2.9–8.7)	(0.5–5.2)
2020	26.9 (11.6–53.0)	53.8 (30.8–87.4)	40.4 (25.9–60.1)	36.9 (23.7–55.0)	24.4 (17.9–32.4)	6.4 (3.9–9.7)	2.1 (0.8–4.6)	2.0 (0.5–5.2)
HMPV								
2017	37.0 (18.5–66.2)	63.9 (38.5–99.8)	50.5 (34.1–72.1)	69.3 (50.5–92.7)	46.7 (37.6–57.4)	5.8 (3.5–9.0)	1.1 (0.2–3.1)	1.0 (0.1–3.6)
2018	67.3 (41.1–103.9)	107.7 (73.7–152.0)	87.5 (65.3–114.7)	92.4 (70.5–118.9)	72.1 (60.6–85.2)	11.5 (8.1–15.8)	3.5 (1.7–6.4)	0.5 (0–2.8)
2019	53.8 (30.8–87.4)	97.6 (65.3–140.1)	75.7 (55.2–101.3)	75.4 (55.8–99.7)	78.9 (66.8–92.5)	11.8 (8.4–16.1)	2.1 (0.8–4.6)	1.5 (0.3–4.4)
2020	0 (0–12.4)	13.5 (3.7–34.5)	6.7 (1.8–17.2)	1.5 (0–8.6)	1.6 (0.3–4.6)	0 (0–1.1)	0.4 (0–2.0)	0.5 (0–2.8)
EV/RV								
2017	434.1 (362.4–515.8)	632.6 (545.4–729.7)	533.3 (476.2–595.4)	511.1 (457.6–569.2)	327.5 (302.9–354.0)	83.2 (73.7–93.7)	22.8 (17.6–29.0)	10.1 (6.2–15.6)
2018	494.6 (417.9–581.4)	551.8 (470.6–643.1)	523.2 (466.7–584.7)	449.5 (399.4–504.1)	377.8 (351.3–406.3)	81.1 (71.7–91.4)	27.7 (21.9–34.5)	7.6 (4.2–12.5)
2019	376.9 (310.3–453.4)	461.0 (387.0–544.9)	418.9 (368.5–474.3)	424.9 (376.2–478.1)	327.5 (302.9–354.0)	91.4 (81.4–102.3)	27.7 (21.9–34.5)	7.6 (4.2–12.5)
2020	60.6 (35.9–95.7)	107.7 (73.7–152.0)	84.1 (62.4–110.9)	95.4 (73.2–122.4)	72.1 (60.6–85.2)	18.5 (14.1–23.7)	9.1 (6.0–13.4)	3.0 (1.1–6.6)

*RSV, respiratory syncytial virus; Adeno, adenovirus; para 1–4, parainfluenza virus types 1–4; HMPV, human metapneumovirus; EV/RV, enterovirus/rhinovirus.
